# Research Advances in the Roles of Circular RNAs in Pathophysiology and Early Diagnosis of Gestational Diabetes Mellitus

**DOI:** 10.3389/fcell.2021.739511

**Published:** 2022-01-04

**Authors:** Yan-ping Zhang, Sha-zhou Ye, Ying-xue Li, Jia-li Chen, Yi-sheng Zhang

**Affiliations:** ^1^ The Affiliated Lihuili Hospital, Ningbo University, Ningbo, China; ^2^ Medical School, Ningbo University, Ningbo, China; ^3^ Translational Research Laboratory for Urology, the Key Laboratory of Ningbo City, Ningbo First Hospital, Ningbo, China

**Keywords:** gestational diabetes mellitus, circRNA, early diagnosis, GDM, pathophysiology

## Abstract

Gestational diabetes mellitus (GDM) refers to different degrees of glucose tolerance abnormalities that occur during pregnancy or are discovered for the first time, which can have a serious impact on the mother and the offspring. The screening of GDM mainly relies on the oral glucose tolerance test (OGTT) at 24–28 weeks of gestation. The early diagnosis and intervention of GDM can greatly improve adverse pregnancy outcomes. However, molecular markers for early prediction and diagnosis of GDM are currently lacking. Therefore, looking for GDM-specific early diagnostic markers has important clinical significance for the prevention and treatment of GDM and the management of subsequent maternal health. Circular RNA (circRNA) is a new type of non-coding RNA. Recent studies have found that circRNAs were involved in the occurrence and development of malignant tumors, metabolic diseases, cardiovascular and cerebrovascular diseases, etc., and could be used as the molecular marker for early diagnosis. Our previous research showed that circRNAs are differentially expressed in serum of GDM pregnant women in the second and third trimester, placental tissues during cesarean delivery, and cord blood. However, the mechanism of circular RNA in GDM still remains unclear. This article focuses on related circRNAs involved in insulin resistance and β-cell dysfunction, speculating on the possible role of circRNAs in the pathophysiology of GDM under the current research context, and has the potential to serve as early molecular markers for the diagnosis of GDM.

## Introduction

Gestational diabetes mellitus (GDM) is defined as varying degrees of glucose intolerance identified for the first time during pregnancy (American Diabetes Association, 2019). In the past decade, with the increasing numbers of pregnant and parturient women who are obese or of advanced age, the incidence of GDM has increased worldwide (McIntyre et al., 2019). According to the latest data from the International Diabetes Federation in 2019 (International Diabetes Federation, 2019), the global prevalence of diabetes in pregnancy is 15.8%, affecting approximately 20.4 million women and newborns each year, with GDM accounting for 83.6% of these cases. In China, the incidence of GDM is 14.8% (Gao et al., 2019). GDM is associated with adverse pregnancy outcomes and long-term complications for the mother and fetus, and has certain effects on the long-term health management of postpartum mothers. Long-term exposure to a hyperglycemic uterine environment can lead to epigenetic changes for the fetus (Franzago et al., 2019), and increase the risk of metabolic and cardiovascular diseases in the offspring (Scholtens et al., 2019; Perak et al., 2021). Recent research demonstrates a 5–10 times higher risk of type 2 diabetes mellitus (T2DM) after childbirth in women with GDM than in women without GDM, with the highest risk at 3–6 years after childbirth (Song et al., 2018; Vounzoulaki et al., 2020).

The current diagnosis of GDM is based on the criteria defined by the International Association of Diabetes and Pregnancy Study Groups. The oral glucose tolerance test has some limitations, including repeated blood sampling, poor compliance of subjects and late diagnosis, because it is impossible to screen high-risk groups of GDM in the first trimester, However, those women with gestational diabetes have abnormal glucose metabolism before OGTT screening (Yefet et al., 2020). Early intervention for GDM, especially lifestyle modification, can effectively reduce fetal exposure to hyperglycemia in the uterus and greatly improve adverse pregnancy outcomes (Ehrlich et al., 2021). Therefore, looking for a specific early diagnostic marker to facilitate early, active, and appropriate intervention is of great significance for preventing and delaying the development and progression of long-term maternal diabetes in the early stage.

Non-coding RNAs, which do not encode proteins, include microRNAs (miRNAs), long non-coding RNAs (lncRNAs), circular RNAs (circRNAs), tRNA-derived small RNAs, and PIWI-interacting RNAs (Slack and Chinnaiyan, 2019). Non-coding RNAs such as miRNAs, lncRNAs, and circRNAs have been reported to serve as potential molecular markers for metabolic diseases, including GDM. Despite the large amount of research on miRNAs and lncRNAs in GDM, little is known about the role of circRNAs in this context. CircRNAs, which are formed by covalent joining of the 3′ and 5′ ends through exon or intron circularization, are more stable than linear RNA (Kristensen et al., 2019). In this review, we summarize recent research advances that have further elucidated the roles of circRNAs in the development and progression of GDM.

## Pathophysiological Features of GDM

In a normal pregnancy, as the gestational age increases, the energy requirements of the fetus increase, and the mother’s basal metabolism increases (Catalano et al., 1999). Insulin resistance is an adaptive response, which promotes increased levels of glucose and free fatty acids in the maternal blood that can be transferred as sources of energy to the fetus (Plows et al., 2018). Although insulin response has increased, insulin secretion is still relatively insufficient. On the other hand, exogenous insulin sensitivity, defined as the ability of insulin to increase glucose uptake in skeletal muscle and adipose tissue, is reduced by about 50% in late pregnancy (Catalano et al., 1991). In addition, placenta, skeletal muscle, and adipose tissue produce factors related to insulin resistance, such as Human placental lactogen (hPL), human placental growth hormone (hPGH), leptin, TNF-α, etc., which may reduce the efficiency of glucose uptake or cause insulin signal transduction obstacle (Kirwan et al., 2002; Andersson-Hall et al., 2020). thus resulting in a progressive reduction in insulin sensitivity. Adenosine monophosphate (AMP)-activated kinase (AMPK), which is decreased in insulin resistant states (Hegarty et al., 2009). The expression of AMPK is decreased in the skeletal muscle and adipose tissue of pregnant women with GDM (Cantó et al., 2010). The elevated blood glucose levels stimulate a compensatory increase in the release of insulin, and there is an adaptive increase in pancreatic β-cell mass caused by increased cell proliferation and reduced apoptosis (Plows et al., 2018).

Potential metabolic abnormalities in gestational diabetes are insulin resistance and pancreatic β-cell dysfunction (Filardi et al., 2020). Insulin resistance in GDM is mediated by various factors, Compared with those in healthy pregnant women, glucose uptake is reduced by approximately half, and insulin sensitivity is decreased approximately 60% in patients with GDM (Catalano, 2014). Peripheral insulin resistance plays an important role in the pathophysiology of GDM. Some women have peripheral insulin resistance before pregnancy or early pregnancy, but it is asymptomatic and undetected. Moreover, in the second and third trimesters that GDM occurs may be due to increased insulin resistance (Catalano et al., 1999). Women with normal glucose tolerance in non-pregnant, insulin binds to the insulin receptor on the cell surface of surrounding tissues (such as skeletal muscle and fat) to activate the tyrosine kinase domain of the insulin receptor β subunit (IRβ), then activates the typical insulin-signaling cascade that induces the redistribution of glucose transporter type 4 (GLUT4) to the cell surface, allowing the cell’s glucose uptake (Tokarz et al., 2018). In pregnant women, the insulin-stimulated glucose transport rate dropped by about 40%, while in GDM women it reduced by 60% (Barbour et al., 2007). The total abundance of GLUT4 in the skeletal muscle did not change in normal pregnant women and GDM patients. Decreased glucose transport was associated with decreased Insulin receptor substrate-1(IRS-1) tyrosine phosphorylation (Barbour et al., 2007). However, in GDM subjects, the decrease of insulin receptor β subunit tyrosine phosphorylation level is related to the further decrease of glucose transport activity (Friedman et al., 1999). The increase in adipose tissue and impaired insulin signal transduction increase insulin resistance by approximately 56%, while progesterone can lower the expression of IRS-1 to inhibit glucose uptake by adipocytes (Nguyen-Ngo et al., 2019). In addition, endoplasmic reticulum stress or mitochondrial dysfunction in skeletal muscle, placental DNA methylation, and activation of adipose tissue inflammation are potential mechanisms of insulin resistance in GDM (Boyle et al., 2013; Lappas, 2014; Liong and Lappas, 2016; Hivert et al., 2020).

Pancreatic β-cells, which are the only insulin-secreting cells in the human body, are critical for blood glucose homeostasis. A reduction in the number and function of β-cells will lead to insufficient insulin release and increased blood glucose levels (Marchetti et al., 2017) Under normal pregnancy, the number of pancreatic islet β cells in women increases adaptively by 1.2∼2.4 fold (Assche et al., 1978; Butler et al., 2010). In the context, GDM is regarded as β-cell dysfunction and cannot compensate for insulin resistance during pregnancy (Xiang et al., 2013). The mechanism of β-cell dysfunction in GDM is not fully understood. Pre-pregnancy obesity, mother’s overnutrition, excessive weight gain during pregnancy, and genetic susceptibility may all cause β-cell dysfunction and hyperglycemia (Moyce and Dolinsky, 2018). The pregnancy hormones prolactin (PrL) and placental prolactin (PL) signal through the prolactin receptor (PRLR) and contribute to the beta cell adaptive response during pregnancy (Baeyens et al., 2016). In GDM, inactivation of prolactin receptors on the surface of islet β cells promotes apoptosis and inhibits proliferation of β cells (Banerjee et al., 2016). Moreover, down-regulation of prolactin action in non-beta cells during pregnancy negatively secondary affects beta cell gene expression and increases beta cell susceptibility to external injury (Shrivastava et al., 2021). Adiponectin plays an important role in the proliferation of beta cells during pregnancy by promoting prolactin expression in trophoblast cells (Qiao et al., 2021). Melatonin deficiency impairs pancreatic remodeling via apoptosis and proliferation of pancreatic islets and impairs beta cell glucose-induced insulin release during pregnancy and lactation (Gomes et al., 2021). In addition, under the mediation of obesity and systemic inflammation, a variety of cytokines (such as tumor necrosis factor α, interleukin-1β and interferon-γ are released, which induces β cells to undergo oxidative stress and endoplasmic reticulum Stress and mitochondrial damage interfere with cell dedifferentiation and cause cell apoptosis (Moyce and Dolinsky, 2018).

## CircRNAs

### Overview of circRNAs

**FIGURE 1:**
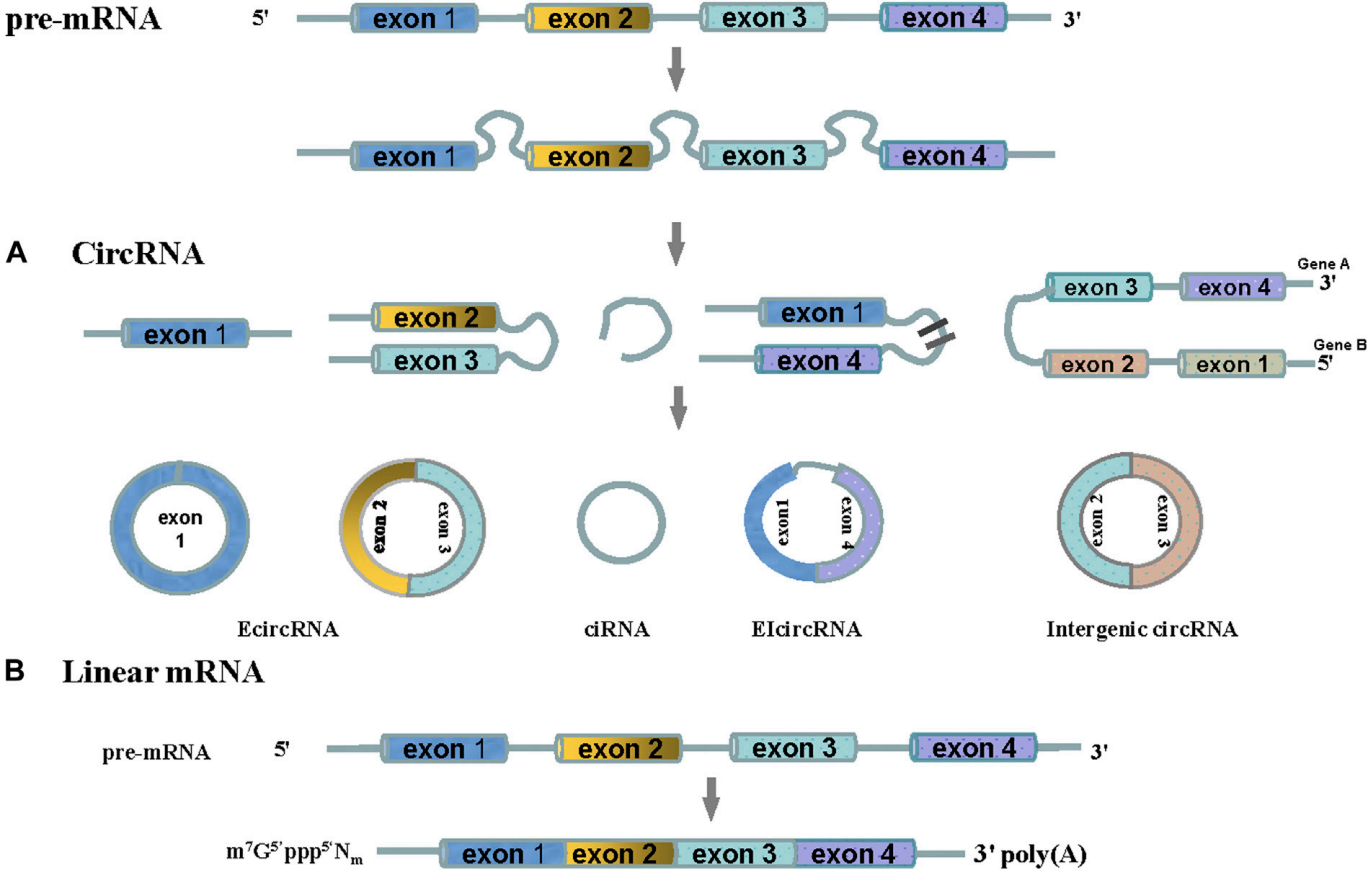
The type of circRNAs. CircRNAs are derived from precursor mRNA. **(A)** According to different biogenesis patterns from genomic regions, circRNAs can be divided into four categories: (1) Exonic circRNA (ecircRNA), ecircRNAs are predominantly generated from the back-splice exons (exon 1 or exon 2 and exon 3), where downstream 3 splice donors are covalently linked to upstream five splice acceptors in reverse order; (2) Circular intronic RNA (ciRNAs), intronic lariats that escape from debranching can lead to the formation of ciRNAs; (3) Exon-intron circRNAs(EIcircRNA), exons 3 and exon 4 are reversed spliced to form a loop containing an intron; (4) Intergenic circRNAs, the exons 3 from gene A and exons 2 from gene B were reversed spliced to form a loop. **(B)** A “cap” (m7G5'ppp5'Nm) was added to the 5 ‘end of pre-mRNA, and a “tail” Q18 (polyA) was added to the 3' end of precursor RNA to form linear RNA.

CircRNAs are created by a back-splicing process in which the downstream splice donor site is joined to the upstream splice acceptor site to form a covalently closed transcript. These molecules were first identified in a plant virus in 1976 (Sanger et al., 1976) and identified as an endogenous RNA splicing product in eukaryotes in 1979 (Hsu and Coca-Prados, 1979). CircRNAs were later found in the human hepatitis D virus in 1986 (Kos et al., 1986). In 2012, Salzman *et al.*(Salzman et al., 2012) analyzed the types and abundance of circRNAs in mammalian cells, and found that more than 10% of expressing genes could produce circRNAs. Based on the genomic source and sequence composition, circRNAs are classified into four types: exonic circRNAs derived from exons, intronic circRNAs derived from introns, exon-intron circRNAs containing both exons and introns, and intergenic circRNAs (Wang et al., 2016; Kristensen et al., 2019; Figure 1).

CircRNAs can escape degradation by exoribonucleases and the half-life of these molecules is 4.8 times longer than that of linear RNA (Jeck and Sharpless, 2014). CircRNAs are also more stable than linear RNAs (Stoll et al., 2020). CircRNAs are abundant in exosomes, and ubiquitous in extracellular fluids including saliva, blood, and urine (Hutchins et al., 2021; Wang et al., 2021a; Xiao et al., 2021). Their expression levels are 10-fold higher than those of linear mRNAs, and are highly conserved in the evolution of species (Jeck et al., 2013). CircRNA expression is time- and space-specific, showing distinct expression patterns in different tissues, cell types and developmental stages (Salzman et al., 2013).

In recent years, there have been significant advances in our understanding of the function of circRNAs. Accumulating evidence shows that circRNAs are the main factors that control gene expression by regulating transcription and translation. Rich in miRNA binding sites, circRNAs can act as a miRNA sponges, or competing endogenous RNAs (Hansen et al., 2013). By binding to RNA binding proteins, circRNAs can alter splicing patterns or mRNA stability, and inhibit the activity of proteins (Conn et al., 2015). CircRNAs can also serve as templates for the translation of proteins, and become a source of pseudogenes that regulate gene expression (Liu and Chen, 2021). However, the molecular mechanisms of the various biological functions of circRNAs remain to be fully elucidated.

### CircRNAs as Novel Molecular Diagnostic Markers

With the recent advances in gene sequencing and bioinformatics technologies, circRNAs have been found to play an important role in various diseases such as cancer, metabolic diseases (*e.g.*, T2DM and hyperlipidemia) as well as disorders of the cardiovascular and immune systems (Lei et al., 2020), (Garikipati et al., 2019). In malignant tumors including gastric cancer, liver cancer, breast cancer, lung cancer, bladder cancer, and cervical cancer, circRNAs are abnormally expressed, and regulate tumor proliferation and invasion, thus representing potential molecular markers for diagnosis and outcome prediction and also as therapeutic targets (Li et al., 2020) (Table 1). Furthermore, numerous studies have indicated that circRNAs are key molecules in metabolic homeostasis, regulate multiple genes related to glucose and lipid metabolism, and mediate the development and progression of metabolic disease. Thus, circRNAs are also implicated as promising targets for resolving metabolic disturbance as well as novel molecular diagnostic markers (Zeng et al., 2021).

**TABLE 1:** CircRNAs as novel biomarkers in human diseases.

Disease	CircRNA name	Sample	Expression	Function	References
		(*n*)			
Pre-diabetes	hsa_circ_0054633	plasma	Up	--	Zhao et al., (2017)
	hsa_circ_0068087	(*n*=40)			
	hsa_circ_0018508				
	CDR1as	plasma	Up	Related to HbA1c	Rezaeinejad et al. (2021)
		(*n* = 200)			
Type 2 diabetes mellitus	hsa_circ_0054633	plasma	Up	Related to glycosylation index	Zhao et al., (2017)
	hsa_circ_0068087	(*n*=40)			
	CircHIPK3	plasma	Up	Related to HbA1c	Rezaeinejad et al. (2021)
		(*n* = 200)			
	hsa-circRNA11783-2	plasma	Down	--	Li et al. (2017)
		(*n* = 124)			
	has_circ_0063425 hsa_circ_0056891	plasma	Up	Related to HbA1c and HOMA-IR	Lu et al. (2021)
		(*n* = 60)			
	hsa_circ_007,110	plasma	Up	Related to hyperlipidemia	Yingying et al. (2021)
	hsa_circ_0071271	(*n* = 206)			
Type 1 diabetes mellitus	hsa_circ_0060450	Plasma	Up	Involved in pancreatitis	Yang et al. (2020a)
		(*n* = 40)			
	circPPM1F	plasma	Up	Regulate islet function	Zhang et al. (2020)
		(*n* = 8)			
Gestational diabetes mellitus	hsa_circ_0005243	Placenta	Down	trophoblast cell dysfunction	Wang et al. (2020a)
		Plasma (in the third trimester)			
		(*n* = 40)			
	hsa_circRNA_102682	Plasma	Down	regulate lipid metabolism	Wu et al. (2021)
		(in the third trimester) (*n* = 200)			
	hsa_circ_0054633	Placenta	Up	Related to glycosylation index	Wu et al. (2019)
		Plasma (in the second/third trimester) (*n* = 130/80)			
	hsa_circ_063981	Plasma	Down	--	Wu et al. (2019)
		(in the third trimester) (*n* = 40)			
	hsa_circ_102893	Plasma (in the first/second trimester)	Down	--	Yang et al. (2020b)
		(*n* = 24/36)			
	circ_5824	Placenta	Down	--	Wang et al., (2019)
	circ_3636	(*n*=45)			
	circ_0395				

## CircRNAs and GDM

CircRNA is involved in insulin resistance and pancreatic β-cell dysfunction (Zhang et al., 2021; Lin et al., 2021), provides certain insights for exploring the pathogenesis of GDM, and provides a possible future direction for the diagnosis of GDM and the prediction of complications.

### CircRNAs and Insulin Resistance

The expression patterns of circRNAs in patients with GDM show distinct differences compared with healthy individuals (Yan et al., 2018). The predicted target genes of circRNAs are involved in glucose and lipid metabolism in GDM. AMPK signaling pathway is involved in the pathophysiology of GDM (Han et al., 2020), AMPK is critical for energy homeostasis, but its activity is decreased in the context of increasing hyperglycemic stress as the gestational age increases (Kumagai et al., 2018). In the skeletal muscle induced by the bacterial endotoxin lipopolysaccharide (LPS) and the pro-inflammatory cytokine IL-1β, activating AMPK could ameliorate inflammation and insulin resistance in GDM (Kumagai et al., 2018). CircACC1, a circRNA derived from the human acetyl-CoA carboxylase 1 gene, is involved in the formation of AMPK complex. Under metabolic stress, circACC1 activates the AMPK signaling pathway through JNK signaling, and promotes fatty acid β-oxidation and glycolysis in cells (Li et al., 2019). Unfortunately, the role of CircACC1 in GDM requires to be verified by further experiments. PAPPA is a metalloproteinase secreted from the human placenta that regulates the bioavailability of insulin-like growth factor (IGF) through the hydrolysis of IGF binding proteins (IGFBPs)-2, 3, and 5 (Boldt and Conover, 2007). *In vitro*, recombinant PAPPA has been shown to stimulate human adipose tissue to expand in an IGFBP-5 and IGF-1-dependent manner (Gyrup and Oxvig, 2007). Rojas–Rodriguez *et al*. (Rojas-Rodriguez et al., 2020) reported that insulin resistance is closely associated with endothelial dysfunction (Aziz et al., 2018), and hsa_circ_0010283 is involved in vascular smooth muscle cell dysfunction by regulating the miR-133a-3p/pregnancy-associated plasma protein A (PAPPA) pathway (Feng et al., 2020). PAPPA-deficient mice develop insulin resistance in pregnancy. Moreover, a study of 6,361 pregnant women demonstrated that the concentration of PAPPA in the circulation is negatively correlated with blood glucose levels and the incidence of GDM (Rojas-Rodriguez et al., 2020). As a miR-149-5p sponge, hsa_circ_0124644 regulates the expression of PAPPA indirectly, resulting in endothelial cell injury (Wang et al., 2020b). The circRNA SCAR is downregulated in liver fibroblasts, and is negatively correlated with insulin resistance (Zhao et al., 2020). CircHIPK3(one of the most abundant circRNAs present in β-cells (Stoll et al., 2018)) regulates hyperglycemia and insulin resistance by increasing the mRNA levels of two key enzymes in glucose metabolism. However, it is uncertain whether these circRNAs have the same regulatory mechanism in peripheral insulin resistance. The circRNA SAMD4A regulates fat formation via the miR-138-5p/EZH2 axis in obesity (Liu et al., 2020), which is a high-risk factor for GDM. Nevertheless, the role of these circRNAs in insulin resistance in GDM merits further investigation.

### CircRNAs and β-Cell Dysfunction

CircRNAs are abundant in human pancreatic islets, and highly conserved between species. As novel regulators for β-cell activity, circRNAs are involved in β-cell dysfunction in hyperglycemia. CircRNA-CDR1as was the first circRNA studied in pancreatic islet cells, and is predominantly located in the cytoplasm (Hansen et al., 2011). The researchers found that CircRNA-CDR1 acts as a miR-7 sponge, regulating insulin secretion, pancreatic β cell proliferation and insulin signal transduction (Xu et al., 2015). Since then, researchers have continued to explore the role of circRNA in beta cell dysfunction. Using microarrays, Stoll L *et al.*(Stoll et al., 2018) found circHIPK3 regulates the apoptosis, proliferation, and insulin secretion of β-cells through sponging miR-124-3p and miR-338-3p and regulating the expression of key genes such as *SLC2A2*, *AKT1*, and *MTPN* (Brozzi and Regazzi, 2021)*.* As shown above, diminished levels of circHIPK3 and CircRNA-CDR1as cause β cells to be unable to release enough insulin to meet the organism’s needs. CircRNA is involved in β cell dysfunction mediated by lipotoxic and pro-inflammatory cytokines (Zhang et al., 2021; Wang et al., 2021b). Wu *et al.*(Wu et al., 2020) revealed that circTulp4 regulated β-cell proliferation to lipotoxicity via the miR-7222-3p/soat1/cyclinD1 signaling pathway. In addition, Stoll et al. (Stoll et al., 2020) discovered an intron circular RNA in mice, rats, and human pancreatic islets—ci-Ins2/ci-INS, which was specifically expressed in pancreatic β cells. It regulated insulin-releasing genes by interacting with RBP TDP-43. Meanwhile, ci-INS was found significantly lower in the islets of type 2 diabetes and to be inversely correlated to HbA1c levels. Sun et al. (Sun et al., 2021) found hsa_circ_0,054,633 mediates apoptosis and insulin secretion via miR-409-3p/caspase-8 axis in human pancreatic β cells. Under the premise that the pathogenesis of GDM is not clear, the reduction in β-cell mass adaptability in patients with GDM may be related to circRNA-mediated regulation of β-cell function and insulin secretion disorder.

### CircRNAs as Molecular Marker for GDM

CircRNA plays an important role in early diagnosis of GDM, assessment of placental function, and prediction of complications. In order to explore the value of circRNA in the diagnosis of GDM, our team found that circRNAs such as hsa_circ_0054633, hsa_circ_103410, hsa_circ_063981, and hsa_circ_102682 were differentially expressed in the placenta, cord blood and maternal plasma during the second and third trimester in women with GDM. At the same time, hsa_circ_0054633 was highly correlated with glycosylated hemoglobin (Wu et al., 2019) and hsa_circ_102682 was closely related to lipid metabolism in GDM(Wu et al., 2021). Nonetheless, if circular RNA is used as a marker for early diagnosis of GDM, whether it changes before OGTT diagnosis in the second trimester. And it is also unclear whether expression in placenta is related to expression in early pregnancy, more experiments are required to explore this. Then, Wang *et al.* (Wang et al., 2020a) discovered hsa_circ_0005243 was significantly downregulated in the placenta and plasma in the third trimester of patients with GDM, and its downregulation induced trophoblast dysfunction and inflammation in a hyperglycemic environment via the β-catenin and NF-κB pathways. Similarly, the dynamic changes of the circular RNA during pregnancy and its value as a molecular marker for early diagnosis or predicting placental functionstill also need to be verified. Increasingly, more and more circRNAs are identified to be expressed differently in the serum of GDM women during the third trimester of pregnancy and the placental tissues during delivery. Meanwhile, the mechanism of action of the corresponding circRNA is gradually being explored. Wang *et al.* (Wang et al., 2019) identified 8,321 circRNAs in GDM and normal placenta tissues, and revealed significantly reduced expression of hsa_circ_5824, hsa_circ_3636, and hsa_circ_0395 in the GDM group. Moreover, the reduced expression of hsa_circRNA_0395 in the placenta of patients with GDM overlapped with the expression of the gene encoding PAPPA2, which can be used to predict the development of fetal macrosomia in GDM. Yang *et al.*(Yang et al., 2020b) found that hsa_circRNA_102893 was downregulated in the plasma of women with GDM in the 15–24 weeks and bound to miR-33a-5p, miR-2115-3p, miR-197-3p, miR-5187-5p, and miR-198 to regulate the expression of 24 target genes. In addition, it was predicted that hsa_circRNA_102893 contained six flanking region binding proteins, including EIF4A3 protein (involved in promoter translation) and AGO protein (involved in the processing and maturation of small RNAs). Tang *et al.*(Tang et al., 2020)found various differentially expressed circRNAs in the placental tissue of patients with GDM by transcriptome sequencing. GO and KEGG pathway analyses indicated that the main function of the circRNAs was to activate phospholipase C and regulate insulin secretion. Furthermore, the circRNAs had one or more target sites for specific binding of miRNAs that facilitated regulation of the development and progression of GDM via the sponge mechanism. Although the abnormal expression of these circRNAs were found, at which stage of pregnancy circRNA changes, whether even occurs before pregnancy, and how they will change after delivery, these string of problems are still unknown at present, and more experiments are needed to clarify *in vivo* and *in vitro*.

## Prospects

Although circRNAs are a focus of research in many fields, their exact roles and regulatory mechanisms in GDM are preliminary remain to be fully elucidated. In-depth and systematic investigations are required to address the specific location of GDM-related circRNAs in cells and the signaling pathways by which they interact with proteins and miRNAs. Based on their characteristic stability and abundance as well as their biological functions, circRNAs show promise as molecular markers for early diagnosis of GDM and prediction of its complications, which will facilitate early intervention for GDM to reduce adverse perinatal outcomes. CircRNAs are also implicated as molecular markers for predicting the long-term risk of GDM and identifying patients at high risk to deliver intervention and guidance to provide a better long-term quality of life.
